# Feeding *Bacillus subtilis* ATCC19659 to Broiler Chickens Enhances Growth Performance and Immune Function by Modulating Intestinal Morphology and Cecum Microbiota

**DOI:** 10.3389/fmicb.2021.798350

**Published:** 2022-02-22

**Authors:** Taha M. Mohamed, Weizhong Sun, Gifty Z. Bumbie, Abdelmotaleb A. Elokil, Khaled Abuelezz Fouad Mohammed, Rao Zebin, Ping Hu, Liuting Wu, Zhiru Tang

**Affiliations:** ^1^Laboratory for Bio-Feed and Molecular Nutrition, College of Animal Science and Technology, Southwest University, Chongqing, China; ^2^Department of Animal and Fish Production, Faculty of Agriculture (Saba Basha), Alexandria University, Alexandria, Egypt; ^3^Animal Production Department, Faculty of Agriculture, Benha University, Moshtohor, Egypt; ^4^Department of Poultry Production, Faculty of Agriculture, Assiut University, Assiut, Egypt

**Keywords:** growth performance, immune response, intestinal morphology, cecum microbiota, broiler, *Bacillus subtilis* ATCC19659

## Abstract

This study investigated dietary supplementation with *Bacillus subtilis* (*BS)* ATCC19659 on growth performance, biochemical indices, intestinal morphology, and cecum microflora in broiler chicks. A total of 600 Arbor 1-day Acres broilers of either sex were allotted to 5 treatments: chicks were fed a corn- and soybean-based diet (CON); chicks were fed basal diet containing 500 mg ZnB/kg (ZnB); chicks were fed basal diet containing 1 × 10^8^ CFU/g feed of BS-ATCC19659 (BS-1); chicks were fed basal diet containing 3 × 10^8^ CFU/g feed of BS-ATCC19659 (BS-3); and chicks were fed basal diet containing 5 × 10^8^ CFU/g feed of BS-ATCC19659 (BS-5). Each treatment comprised 6 replicates with 20 birds for each replicate pen. Chicks in the BS-5 and BS-3 groups had higher body weight at the 21^st^ and 42^nd^ days and average daily gain from 1 to 21 days than that in the CON group (*p* < 0.05). Chicks in the BS-5 and ZnB groups had higher serum antioxidant activities and immunity response than those in the CON group (*p* < 0.05). Compared with the CON group, the liver mRNA abundance of *GHR*, *TGF-β*, *IGF-1*, *IFN-γ*, *SOD*, *CAT*, and *GPX* of chicks in three *BS* groups and the ileum villus length (μm) of chicks in BS-3 and ZnB groups was increased (*p* < 0.05). Compared with the CON group, the villus height-to-crypt depth ratio of the ileum of chicks in the BS-5 and BS-3 groups and the crypt depth and villus height-to-crypt depth ratio of the jejunum in the BS-5 and ZnB groups were increased (*p* < 0.05). The abundance of the *Cyanobacteria* phyla in the cecum decreased in response to treatment with both *BS*-ATCC19659 and ZnB groups (*p* < 0.05). Compared with the CON group, the cecum abundance of genera *GCA-900066575* (*Lachnospiraceae*), *Anaerofustis*, and *Papillibacter* (*Firmicutes* phylum) in three *BS* groups were increased (*p* < 0.05); The abundance of genus *Escherichia–Shigella* reduced in the BS-3 group (*p* < 0.05). Compared with the CON group, the cecum abundance of genus *Clostridia_unclassified* in ZnB and BS-5 groups was decreased (*p* < 0.05) of broilers. Generally, *Bacillus subtilis* ATCC19659 as feed additive positively affected growth performance, immunity response, and cecal microflora of broilers.

## Introduction

Antibiotics were used to eliminate poultry pathogens and promote growth performance and improve egg production. However, because of the potential of antibiotic-resistant strains of pathogenic organisms spreading into the environment, human infection through the food chain can lead to serious consequences to public health (Singer et al., 2003). Limiting antibiotics as growth enhancers in livestock production prompted a need to evaluate and present new alternatives to growth promoters (Tang et al., 2017). Indeed, this is primarily to avoid pathogenic bacterial resistance to antibiotics and to meet the growing consumer consciousness about hazardous residues in poultry meat and eggs. On the other hand, banning antibiotics in the poultry industry would result in reduced profits due to reduced flock productivity (Yang et al., 2019). Therefore, some viable alternatives to dietary antibiotics, such as probiotics, prebiotics, and organic acids, have been suggested in this regard to promote healthy flock productivity (Diaz-Sanchez et al., 2015; Suresh et al., 2018). Compared to antibiotics, these alternatives were characterized by natural environmental safety, no harmful residues accumulating in poultry eggs and meat, promoting a healthy gut microbiota and improving feed intake, feed conversion, and growth rate (Gadde et al., 2017).

Zinc bacitracin (ZnB) is an antibiotic commonly used in dietary commercial broiler production. Zinc bacitracin is a combination of high molecular weight polypeptides produced by *Bacillus licheniformis*, considered a growth promoter (Pedroso et al., 2006; Crisol-Martínez et al., 2017). *Bacillus subtilis* is commonly included in probiotic supplement formulations as a healthy probiotic strain to improve growth performance and enhance immune and digestive system health (Bai et al., 2017). It consumes a high amount of free oxygen during colonization in the gut, restricting the growth of pathogenic aerobic bacteria to enhance the growth of anaerobic bacteria such as *Lactobacillus* and *Bifidobacterium* (Gao et al., 2017). Previous studies demonstrated that dietary supplementation of *Bacillus subtilis* improved growth performance (Amerah et al., 2013; Jayaraman et al., 2017; Wang et al., 2017), enhanced immune functions (Rajput et al., 2013; Lee et al., 2014; Bai et al., 2017; Liu et al., 2019), increased the absorption of nutrients through a positive influence on intestinal morphology (Frizzas de et al., 2003; Palamidi et al., 2016; Zhang et al., 2016; He et al., 2019), improved antioxidant capacity (He et al., 2019; Liu et al., 2019), and affected the composition of the broiler microflora (Hong et al., 2019; Li et al., 2019). Lee et al. (2014) reported that supplementing dietary *Bacillus subtilis* in pigs significantly affected microbiota compositions and improved immune function.

Gut microbiota (GM) is the totality of microorganisms of bacteria, viruses, protozoa, and fungi that have colonized the gastrointestinal tract of the host. It regulates energy harvest from diet, intestinal immunity, and metabolic performance to identify the host productivity (Elokil et al., 2020a). GM could be modified by numerous techniques, including administration of synbiotics, probiotics, antibiotics, prebiotics, and fecal transplantation (Cammarota et al., 2014; Elokil et al., 2020b).

This present study was conducted to investigate *Bacillus subtilis* ATCC19659 (*BS*-ATCC19659) as a substitute for antibiotics in poultry diets. Therefore, this study aimed to elucidate host–microbiome interactions under the influence of dietary supplementation at three different concentrations of *BS*-ATCC19659 on the host performance of broiler (growth, antioxidant status, immunity, and gastrointestinal responses) and the intestinal microbial community.

## Materials and Methods

### Ethics Statement

Experimental procedures were approved by the License of Experimental Animals (SYXK 2014-0002) of the Animal Experimentation Ethics Committee of Southwest University, Chongqing, China, and birds were raised following the guidelines described by the Animal Care Committee of Chongqing, China. In addition, efforts were made to reduce animal suffering and were carried out in compliance with the “ARRIVE” guidelines for reporting *in vivo* experiments in animal research.

### Bacterial Strain

*Bacillus subtilis* ATCC19659 (*BS*-ATCC19659) was provided from (KWIk-STIk™, Microbiologics, Microbiologics, Inc., Saint Cloud, United States), and total content from *Bacillus subtilis* was prepared of a viable bacterium by serial dilution plate counts after culturing in nutrient agar at 37°C for 14 h under an aerobic environment.

### Experimental Design and Management

Six hundred day-of-hatch Arbor Acres broiler chicks were individually weighed and randomly assigned to five groups with six replicates per group and 20 chicks per replicate. The broiler chickens were fed a corn–soybean-based diet. Birds in the control group (CON group) were fed a basal diet without *BS*-ATCC19659 and antibiotic, those in the ZnB group were fed a basal diet with 500 mg/kg of zinc bacitracin, and *BS*-ATCC19659 groups were fed a basal diet with *Bacillus subtilis ATCC19659* at 1 × 10^8^ CFU/g feed (BS-1 group), 3 × 10^8^ CFU/g feed (BS-3 group), and 5 × 10^8^ CFU/g feed (BS-5 group). Mashed feed and water were provided *ad libitum*, and a constant lighting program was used. The basal diet was formulated to meet NRC (1994) requirements during the starter (1–21 days) and finisher periods (22–42 days). The composition and the calculated nutrient content of the experimental diet are shown in Table 1. Every morning, before the inclusion of *BS*-ATCC19659, all the birds had their water and feed withdrawn for about 3 h. After this period, the feed was mixed with (*BS*-ATCC19659) with different concentrations, and birds consumed them within 30 min. Moreover, birds in CON and ZnB groups consumed a similar amount of their usual feed. Once feed was finished, the regular water and feed were restored to birds. The adding of *BS*-ATCC19659 in feed was in final concentrations of 1, 3, and 5 × 10^8^ CFU/g, as described by Bortoluzzi et al. (2019). All birds were housed in cages of the same size (30 × 175 × 155 cm) at 33°C and reduced by 3°C each week to 24°C until the end of the experiment. At the age of 2 weeks, chicks in all groups were vaccinated against essential Newcastle disease (YEBIO^®^, Qingdao, China) using the LaSota B1 Strain of Newcastle disease virus in live freeze-dried form by adding to their drinking water.

**TABLE 1:** Ingredients and nutrients composition of the basal diets (DM basis).

Ingredients (%)	Starter (1–21 days)	Finisher (21–42 days)
Corn	58.00	63.00
Soybean (44% CP)^a^	28.20	24.90
Gluten (60% CP)^b^	7.00	6.00
Dicalcium phosphate	1.80	1.20
Limestone	1.30	1.30
Soybean oil	2.00	2.00
Salt	0.30	0.30
L-Lysine HCl	0.20	0.20
DL-Methionine	0.20	0.10
Premix^c^	1.00	1.00
Total	100	100

**Calculated composition**		
ME (kcal/kg)^d^	3,012.26	3,068.97
Crude protein (%)	21.99	20.28
Calcium (%)	0.98	0.84
Total phosphor (%)	0.72	0.59
Methionine (%)	0.59	0.47
Methionine + cysteine (%)	0.95	0.80
Lysine (%)	1.14	1.06

*^a,b^Crude protein.*

*^c^Provided the following per kilogram of diet: 13,000 IU of vitamin A; 1,300 IU of vitamin D; 65 IU of vitamin E; 3.4 mg of menadione; 37 mg of pantothenic acid; 6.6 mg of riboflavin; 3.7 mg of folic acid; 39 mg of niacin; 1.0 mg of thiamine; 4.3 mg of vitamin B6; 0.23 mg biotin; 0.075 mg of vitamin B12: 43 mg of choline chloride.*

*170 mg of zinc; 140 mg of iron; 34 mg of manganese; 16 mg of copper; 0.29 mg of iodine; 0.29 mg of selenium. ^d^Metabolizable energy.*

### Growth Performance

Feed intake of chicks and the number of dead birds were recorded daily. Chicks were weighed on days 0, 21, and 42 before feeding in the morning. The average daily feed intake (ADFI), average daily weight gain (ADG), and feed conversion rate (FCR) were calculated based on the following formulas: ADFI (g/d) = total food intake (g)/feed days (d); ADG (g/d) = (final weight - initial weight) (g)/feed days (d); FCR (g/g) = ADFI (g/d)/ADG (g/d). Survivability rates (%) = (Number of surviving birds at the end of experimental period/initial number of birds) × 100.

### Measurements and Sampling

At the end of the experimental period (age 42 days), the six birds with bodyweight close to the average bodyweight from each treatment were selected. Blood samples were collected from the jugular vein in non-heparinized tubes (10.0 ml). The collected blood samples were centrifuged at 3,500 rpm for 10 min at 4°C to harvest the serum. The serum was stored at –20°C for biochemical analysis and ELISA. After blood sampling, the six birds selected from each treatment were anesthetized with an intravenous injection of sodium pentobarbital (50 mg/kg BW) and exsanguinated, and segments of the mid-ileum and jejunum were collected, fixed with formalin for 48 h and paraffin-embedded. A total of five samples from each treatment from both liver and jejunum tissue were immediately collected after slaughtering each group and stored at -80°C until RNA isolation. A total of six samples from each treatment from both ceca were taken out after slaughtering from each group. Then, the cecal digesta samples were immediately collected in a sterilizing tube, frozen in liquid N_2_, and stored at −80°C until further analysis.

### Serum-Biochemical Indexes

The contents of serum malondialdehyde (MDA) and the activities of glutathione (GSH), total antioxidant capacity (T-SOD), catalase (CAT), and superoxide dismutase (SOD) were measured. Samples were analyzed by an autoanalyzer (SHIMADZU CL-8,000 automatic autoanalyzer, Shanghai, China), and commercial kits acquired from Nanjing Jiancheng Bioengineering Institute, Nanjing, China (NJJCBIO),^1^ were used. The serum immunoglobulin M (IgM), immunoglobulin G (IgG), immunoglobulin A (IgA), immunoglobulin E (IgE), secretory IgA (sIgA), complement component 3 (C3), and complement component 4 (C4) were determined using commercial kits by following the manufacturer’s instructions (NJJCBIO). In addition, interleukins IL-4, IL-6, IL-10, tumor necrosis factor-α (TNF-α), and transforming growth factor-beta (TGF-β) in serum were measured by chicken-specific ELISA kits (NJJCBIO) following the manufacturer’s instructions. The information of all kits is shown in Supplementary Table 1.

### H&E Staining

The jejunum and ileum morphology was analyzed based on the H&E staining method described by Wang et al. (2009). From each sample, two sections (100 μm) were obtained and stained with hematoxylin for 1 min. After that, all sections were counterstained with eosin for 10 s. All these variables were measured with a camera (Olympus; TH4-200; Tokyo, Japan) coupled with computer-assisted digital Image-Pro Plus (IPP) analysis software (Image-Pro Plus 4.5, Media Cybernetics, Silver Spring, MD, United States) to assess the maximum villus length, crypt depth, and submucosa/muscularis/serosa.

### Real-Time RT-PCR

Total RNA was independently extracted from liver and jejunum samples (*n* = 5), using the TRIzol reagent (15596026, Ambion, Chongqing, China), according to the instructions of the manufacturer. Then, RNA was purified with DNase I (TaKaRa, Shiga, Japan) and an RNA clean kit (Tiangen, China). The quality and quantity of RNA were detected using a NanoDrop spectrophotometer (Thermo Scientific, Shanghai, China) at an absorption rate of 260:280 nm and using gel electrophoresis. To synthesize cDNA, total RNA was reverse-transcribed with MMLV reverse transcriptase (M1705, Promega, Chongqing, China), 5ÕM-MLV Buffer (M1705, Promega, Chongqing, China), and RNasin inhibitor (N2115, Promega, Chongqing, China), dNTPs (4030Q, Takara, Chongqing, China).

Quantitative real-time PCR (qRT-PCR) was done to determine the expression of seven target genes of growth hormone receptor (*GHR*), transforming growth factor-beta (*TGF-β*), insulin-like growth factor-1 (*IGF-1*), interferon-γ (*IFN-γ*), superoxide dismutase (*SOD*), catalase (*CAT*) and glutathione peroxidase (*GPX*) using the qTOWER 3G Real-Time PCR System (Lead Scientific Technology, Chengdu, China) with the ChamQ Universal SYBR qPCR Master Mix (Q711-02, Vazyme, Chongqing, China) and performed in a total volume of 20 μl. A separation curve analysis step of 5 tissue samples from each group was amplified in triplicate. The relative gene expression mRNA was determined to those of the housekeeping gene for eukaryotic (β*-actin*). The 2^−ΔΔCt^ method was used to measure mRNA abundance for calculating the Δ Ct value as (Ct_target_-Ct_B–actin_) treatment-(Ct_target_ -Ct_B–actin_) control. The primer information for targeting genes is shown in Supplementary Table 2.

### Analysis of 16S rDNA Gene Sequencing

For DNA extraction, cecum content samples collected on day 42 were beaded with a Mini-BeadBeater, and then the DNA was extracted using the Power Fecal DNA Isolation Kit (MO BIO, Carlsbad, CA, United States). DNA was stained using the Quant-iT Pico Green dsDNA Kit (Invitrogen Ltd., Paisley, United Kingdom) and quantified using a NanoDrop spectrophotometer (Nyxor Biotech, Paris, France).

For DNA MiSeq sequencing, PCR amplification of the V4 region of bacterial 16S rDNA was performed using the universal primers 515F (5′-*CCTACGGGNGGCWGCAG*-*3′) and 80*6R *(5′*-*GGACTACHVGGGTWTCTAAT*-*3′), incorporating the FLX Titanium adapters and a sample barcode sequence. The cycling parameters were as follows: 4 min of initial denaturation at 94°C; 25 cycles of denaturation at 94°C (30 s), annealing at 50°C (45 s), and elongation at 72°C (30 s); and final extension at 72°C for 5 min. Three separate PCR reactions for each sample were pooled for MiSeq sequencing. The PCR products were separated by 1.5*% agarose gel electrophoresis and purified using the QIAquick Gel Extraction Kit (Qiagen, Hilden, Germany). Amplicons were quantified using the Quant-iT Pico Green dsDNA Assay Kit (Invitrogen, Carlsbad, CA, United States). Equal concentrations of amplicons were pooled from each sample. Libraries were constructed using the TruSeq DNA PCR-Free Sample Prep Kit, and MiSeq sequencing was performed with the MiSeq Reagent Kit v2 (Illumina, San Diego, CA, United States).

Based on unique barcodes of samples, paired-end reads were assigned to them and shortened by cutting off the barcode and primer sequence. Merging of paired-end reads was done using FLASH. The resulting sequences were further screened and filtered for quality and length. Under specific conditions of filtering, quality filtering on the raw reads was performed to obtain clean tags that are high in quality according to fqtrim (v0.94). V-search software (v2.3.4) was used in filtering chimeric sequences. Feature table and feature sequence were obtained after dereplication using DADA2. The relative abundance of each sample was normalized using feature abundance; this was done according to the SILVA (release 132) classifier. QIIME2 calculated alpha and beta diversities analysis; the graphs were drawn by the R package.

### Statistical Analysis

Beta diversity analyses for the principal component analysis (PCA), principal coordinate analysis (PCoA), and nonmetric multidimensional scaling (NMDS) were used to obtain the comparative analysis of intergroup and group differences in terms of unique fraction distance. Alpha diversity analyses through five indices of chao1, observed OUT, Goods coverage, Shannon, and Simpson were analyzed by one-way ANOVA analysis, and the differences were compared using Duncan’s test (*p* < 0.05). The permutational analysis of variance (PERMANOVA) analyzed the significant differences in beta diversity. The relative abundances at the phylum and genus were estimated by the Kruskal–Wallis test (*p* < 0.05). Data on growth performance, serum-biochemical indices, intestinal morphology, and gene expression in the liver and jejunum were analyzed using one-way ANOVA and performed using the general linear model (GLM) procedure of SAS (SAS, 2002, Cary, NC, United States, version 9). Individual chicks were considered as experimental units, and one fixed effect was included in the statistical model. All differences were considered significantly different at *p* < 0.05. Pairwise comparisons were performed using Duncan’s multiple-range test.

## Results

### Growth Performance Assessment of Host Broiler

The growth performance results are shown in Table 2. An increase in weight was observed at the 21^st^ and 42^nd^ days of broiler in response to dietary BS-3 and BS-5 groups compared to the CON and ZnB groups (*p* < 0.05). Compared to chicks in the ZnB group, BS-5 and BS-3 groups display a significant effect on ADG during 1–21 days (*p* < 0.05). However, there was no significant effect on ADFI, ADG, FCR, and survivability rates among the five groups (Table 2).

**TABLE 2:** Effect of dietary supplementation of *Bacillus subtilis* ATCC19659 on the broiler’s growth performance.

Items^1^	Treatments^2^					SEM	*p*-value
	CON	ZnB	BS-1	BS-3	BS-5		
**(1–21 days)**							
1 day (g)	47.08	47.32	47.05	47.49	47.14	0.172	0.915
21 day (g)	641.6^bc^	621.31^c^	646.04^abc^	657.36^ab^	670.54^a^	4.164	0.004
ADFI (g/day)	40.13	40.28	42.79	43.85	42.71	0.601	0.185
ADG (g/day)	28.27^bc^	27.28^c^	28.59^ab^	29.07^ab^	29.62^a^	0.22	0.004
FCR (g/g)	1.42	1.48	1.5	1.51	1.44	0.021	0.653
Survivability rates (%)	99.17	98.33	98.33	97.5	99.17	0.49	0.826

**(21–42 days)**							
42 day (g)	1600.39^ab^	1,578.24^b^	1,620.1^ab^	1,652.0^a^	1,636.98^a^	8.305	0.041
ADFI (g/day)	87.67	86.65	89.95	90.57	88.35	0.905	0.664
ADG (g/day)	50.48	50.33	51.37	52.55	51.35	0.555	0.755
FCR (g/g)	1.74	1.73	1.75	1.73	1.72	0.0134	0.96
Survivability rates (%)	96.67	99.17	100	98.33	99.17	0.48	0.225

**(1–42)**							
ADFI (g/day)	62.71	62.30	65.2	66.06	64.39	0.646	0.305
ADG (g/day)	38.82	38.22	39.41	40.22	39.94	0.295	0.189
FCR (g/g)	1.62	1.63	1.65	1.64	1.61	0.01	0.65
Survivability rates (%)	95.83	97.5	98.33	95.83	98.33	0.746	0.710

*^1^ADG, average daily body gain; ADFI, average daily feed intake; FCR (g/g), ADFI (g/day)/ADG (g/day).*

*^2^CON, chicks were fed a corn- and soybean-based diet; ZnB, chicks were fed basal diet containing 500 mg ZnB/kg; BS-1, chicks were fed basal diet containing 1 × 10^8^ CFU/g feed of BS-ATCC19659; BS-3, chicks were fed basal diet containing 3 × 10^8^ CFU/g feed of BS-ATCC19659; BS-5, chicks were fed basal diet containing 5 × 10^8^ CFU/g feed of BS-ATCC19659.*

*^a,b,c^Values in the same row with different letter superscripts mean significant differences (p < 0.05). Data are presented as the mean ± SEM (n = 6).*

### Serum Antioxidant Assessment of Host Broiler

The results of the serum antioxidant profile are shown in Table 3. There were significant improvements in serum activities of SOD, CAT, T-AOC, and GSH in both BS-5 and ZnB groups compared to the CON group (*p* < 0.05). On the other hand, the serum concentration of MDA was significantly decreased in both the BS-5 and ZnB groups (*p* < 0.05) (Table 3). Thus, collectively, the antioxidant defense was improved as affected by the dietary inclusion of *BS*-ATCC19659.

**TABLE 3:** Effect of dietary supplementation of *Bacillus subtilis* ATCC19659 on the broiler’s serum antioxidant indexes, immunoglobulin, and cytokine profile.

Items^1^	Treatments^2^					SEM	*p*-value
	CON	ZnB	BS-1	BS-3	BS-5		
SOD (u/mL)	104.24^c^	140.34^a^	103.94^c^	120.64^b^	138.37^a^	3.440	<0.001
CAT (U/mL)	4.63^b^	8.47^a^	4.86^b^	7.00^a^	7.45^a^	0.347	<0.001
MDA (nmol/mL)	9.18^a^	3.10^c^	8.83^a^	7.97^a^	5.96^b^	0.408	<0.001
T-AOC (U/mL)	6.56^d^	15.46^a^	5.79^d^	8.76^c^	12.45^b^	0.725	<0.001
GSH (mg/gL)	19.29^d^	35.86^a^	22.77^cd^	28.27^bc^	32.09^ab^	1.434	<0.001
IgE (μg/mL)	0.016^c^	0.02^a^	0.017^c^	0.017^c^	0.02^b^	0.001	<0.001
IgA (μg/mL)	0.51^d^	0.71^a^	0.59^c^	0.62^bc^	0.65^b^	0.013	<0.001
IgG (μg/mL)	1.78^c^	2.79^a^	1.94^c^	2.35^b^	2.34^b^	0.39	<0.001
IgM (μg/mL)	0.90^c^	1.22^a^	0.96^c^	1.07^b^	1.09^b^	0.023	<0.001
C3 (mg/mL)	0.59^b^	0.77^a^	0.06^c^	0.67^bc^	0.71^ab^	0.186	0.002
C4 (mg/mL)	0.14^b^	0.19^a^	0.16^ab^	0.17^ab^	0.19^a^	0.006	0.007
sIgA (ng/mL)	1.49^c^	2.17^a^	1.75^bc^	1.84^b^	1.9^ab^	0.056	<0.001
IL-10 (ng/L)	36.13^d^	63.52^a^	43.06^cd^	49.72^bc^	51.89^b^	2.02	<0.001
IL-4 (ng/L)	159.88^a^	103.41^d^	142.87^b^	126.08^c^	115.4^cd^	4.25	<0.001
IL-6 (ng/L)	29.34^a^	16.57^c^	23.7^b^	23.11^b^	19.29^c^	0.923	<0.001
TNF-α (ng/L)	74.67^a^	50.96^c^	74.73^a^	67.06^ab^	59.03^bc^	2.104	<0.001
TGF-β (ng/L)	134.32^c^	195.92^a^	144.13^*c*^	161.29^*b*^	167.16^*b*^	4.44	<0.001

*^1^GSH, glutathione; T-AOC, total antioxidant capacity; MDA, malondialdehyde; CAT, catalase; SOD, superoxide dismutase; IgM, immunoglobulin M; IgG, immunoglobulin G; IgA, immunoglobulin A; IgE, immunoglobulin E; sIgA, secretory IgA; C4, complement component 4; C3, complement component 3; IL-10, interleukin-10; IL-4, interleukin-4; IL-6, interleukin-6; TNF-α, tumor necrosis factor-alpha; TGF-β, tumor necrosis factor-beta. ^2^CON, chicks were fed a corn-and soybean-based diet; ZnB, chicks were fed basal diet containing 500 mg ZnB/kg; BS-1, chicks were fed basal diet containing 1 × 10^8^ CFU/g feed of BS-ATCC19659; BS-3, chicks were fed basal diet containing 3 × 10^8^ CFU/g feed of BS-ATCC19659; BS-5, chicks were fed basal diet containing 5 × 10^8^ CFU/g feed of BS-ATCC19659.*

*^a,b,c,d^Values in the same row with different letter superscripts mean significant differences (p < 0.05). Data are presented as the mean ± SEM (n = 6).*

### Serum Immunity Assessment of Host Broiler

The dietary administration of *BS*-ATCC19659 groups for 42 days on the levels of immunoglobulins, complement components, sIgA, interleukins, TNF-α, and TGF-β in serum of the host broiler is shown in Table 3. ZnB and BS-5 groups significantly increased the levels of immunoglobulins (IgE, IgA, and IgM) and C3, C4, sIgA, IL-10, and TGF-β as compared with that of the CON group (*p* < 0.05); IgG was significantly higher in ZnB and BS-3 groups than those in the CON group (*p* < 0.05). On the other hand, IL-4 and IL-6 were increased significantly in the serum of the CON group compared to both *BS*-ATCC19659 and ZnB groups (*p* < 0.05); TNF-α was significantly higher in CON and BS-1 groups (*p* < 0.05) (Table 3).

### Intestinal Histomorphometric Assessment of Host Broiler

**FIGURE 1:**
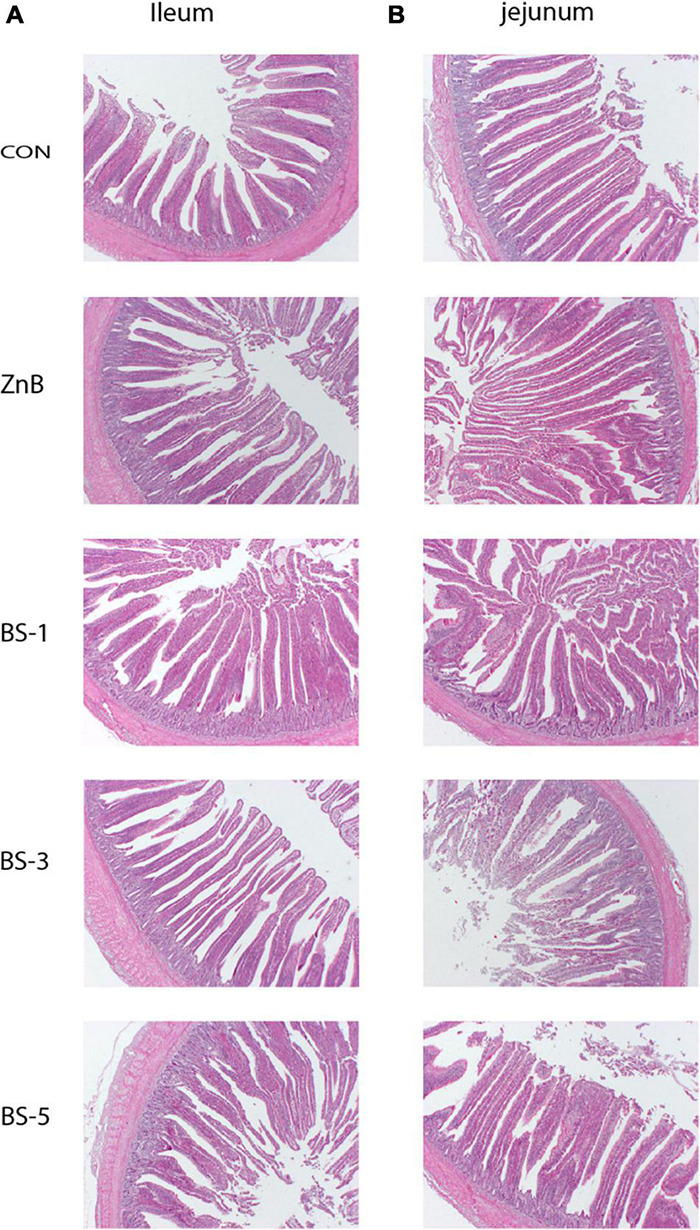
Electron micrograph images of the ileum **(A)** and jejunum **(B)** tissues from broiler treatments. CON, chicks were fed a corn- and soybean-based diet; ZnB, chicks were fed basal diet containing 500 mg ZnB/kg; BS-1, chicks were fed basal diet containing 1 × 10^8^ CFU/g feed of *BS*-ATCC19659; BS-3, chicks were fed basal diet containing 3 × 10^8^ CFU/g feed of *BS*-ATCC19659; BS-5, chicks were fed basal diet containing 5 × 10^8^ CFU/g feed of *BS*-ATCC19659.

The effects of dietary administration of *BS*-ATCC19659 for 42 days on the morphology and morphometry of the ileum and jejunum in broiler are shown in Figure 1. The results showed that the heights of the villus length (μm) in the sections of the ileum were significantly increased with the BS-3 and ZnB groups (*p* < 0.05); the villus/crypt ratio was significantly higher in the BS-5 and BS-3 groups (*p* < 0.05) (Table 4). However, the crypt depth (μm) in the sections of the jejunum was measured in both CON, BS-1, and BS-3 groups (*p* < 0.05); the villus/crypt ratio was significantly increased in the ZnB and BS-5 groups compared to that of the CON group (*p* < 0.05). Conversely, the crypt depth and villus length in the ileum and jejunum were not affected by the treatments, respectively, as shown in Table 4.

**TABLE 4:** Effect of dietary supplementation of *Bacillus subtilis* ATCC19659 on intestinal morphology of the ileum and jejunum in broilers.

Items	Treatments^1^					SEM	*P*-value
	CON	ZnB	BS-1	BS-3	BS-5		
**Ileum**							
Villus length (μm)	598.33^c^	759.20^a^	679.95^bc^	839.46^a^	693.28^abc^	23.32	0.015
Crypt depth (μm)	116.7	124.6	131.45	133.31	118.59	3.767	0.544
Villus height/crypt dept	5.21^b^	6.12^ab^	5.26^b^	7.47^a^	6.78^ab^	0.241	0.009

**Jejunum**							
Villi length (μm)	934.25	1033.17	830.64	843.71	874.92	24.10	0.483
Crypt depth (μm)	152.83^a^	117.06^b^	161.9^a^	152.71^a^	140.27^ab^	4.01	0.004
Villus height/crypt dept	6.53^bc^	9.44^a^	5.21^c^	5.98^bc^	6.89^b^	0.256	<0.001

*^1^CON, chicks were fed a corn-and soybean-based diet; ZnB, chicks were fed basal diet containing 500 mg ZnB/kg; BS-1, chicks were fed basal diet containing 1 × 10^8^ CFU/g feed of BS-ATCC19659; BS-3, chicks were fed basal diet containing 3 × 10^8^ CFU/g feed of BS-ATCC19659; BS-5, chicks were fed basal diet containing 5 × 10^8^ CFU/g feed of BS-ATCC19659.*

*^a,b,c^Values in the same row with different letter superscripts mean significant differences (p < 0.05). Data are presented as the mean ± SEM (n = 6).*

### Growth, Antioxidant, and Cytokine Gene Expression Assessment of Host Broiler

The relative mRNA expression of target genes was compared using qRT-PCR in the liver and intestinal tissues among the treatment groups following the 2^–ΔΔCT^ method and is shown in Table 5. Regarding relative mRNA expression in liver tissue, the highest mRNA expressions for *TGF-β*, *IGF-1*, and *IFN-γ* genes were detected in the BS-3 group compared to the CON group (*p* < 0.05); *GHR* and *SOD* genes were significantly higher in the BS-1 than CON group (*p* < 0.05); *GPX* and *CAT* genes were significantly increased in the BS-5 group compared to the CON group (*p* < 0.05) (Table 5). In the intestinal tissue, the highest mRNA expressions for *GHR*, *TGF-β*, *IFN-γ*, and *GPX* genes were measured in the CON group (*p* < 0.05). Conversely, *the IGF-1* gene was detected in the ZnB and CON groups than in other groups (*p* < 0.05); *CAT* and *SOD* genes were not affected by the dietary supplementation with ZnB and *BS*-ATCC19659 (Table 5).

**TABLE 5:** Effects of dietary supplementation of *Bacillus subtilis* ATCC19659 on the mRNA abundance of *GHR*, *TGF-β*, *IGF-1*, *IFN-γ*, *SOD*, *CAT*, and *GPX* in liver and intestine tissue of broiler.

Treatments^2^	Items^1^						
	*GHR*	*TGF-β*	*IGF-1*	*IFN*-γ	*SOD*	*CAT*	*GPX*
**Liver**							
CON	1.12^b^	1.03^b^	1.04^b^	1.13^b^	1.13^b^	1.09^b^	1.02^b^
ZnB	0.5^c^	1.02^b^	0.99^b^	1.49^b^	1.14^b^	1.65^b^	1.02^b^
BS-1	2.35^a^	0.58^c^	1.34^b^	1.05^b^	8.23^a^	5.01^a^	1.32^a^
BS-3	2.22^a^	1.46^a^	2.12^a^	2.4^a^	7.47^a^	4.81^a^	1.07^b^
BS-5	1.3^b^	1.44^a^	1.88^a^	1.61^b^	1.51^b^	5.45^a^	1.42^a^
SEM	0.16	0.08	0.116	0.123	0.672	0.393	0.047
*p*-value	<0.001	0.001	<0.001	<0.001	<0.001	<0.001	0.002

**Intestinal**							
CON	1.09^a^	1.22^a^	1.1^a^	1.17^a^	1.09	1.09	1.16^a^
ZnB	0.63^b^	0.76^b^	1.17^a^	0.92^b^	0.9	0.84	0.64^b^
BS-1	0.54^b^	0.80^b^	0.45^b^	0.78^b^	1.13	1.2	0.61^b^
BS-3	0.57^b^	0.72^b^	0.59^b^	0.85^b^	0.96	0.77	0.62^b^
BS-5	0.49^b^	0.38^c^	0.44^b^	0.68^b^	1.23	0.8	0.57^b^
SEM	0.049	0.071	0.073	0.05	0.088	0.063	0.07
*p*-value	< 0.001	0.001	< 0.001	0.006	0.717	0.09	0.02

*^1^GHR, growth hormone receptor; TGF-β, transforming growth factor-beta; IGF-1, insulin like growth factor 1; IFN-γ, interferon γ; SOD, superoxide dismutase; CAT, catalase; GPX, glutathione peroxidase.*

*^2^CON, Chicks were fed a corn-and soybean-based diet; ZnB, chicks were fed basal diet containing 500 mg ZnB/kg; BS-1, chicks were fed basal diet containing 1 × 10^8^ CFU/g feed of BS-ATCC19659; BS-3, chicks were fed basal diet containing 3 × 10^8^ CFU/g feed of BS-ATCC19659; BS-5, chicks were fed basal diet containing 5 × 10^8^ feed CFU/g of BS-ATCC19659.*

*^a,b,c^Values in the same row with different letter superscripts mean significant differences (p < 0.05). Data are presented as the mean ± SEM (n = 5).*

### Microbial Diversity Assessment of Host Broiler

**FIGURE 2:**
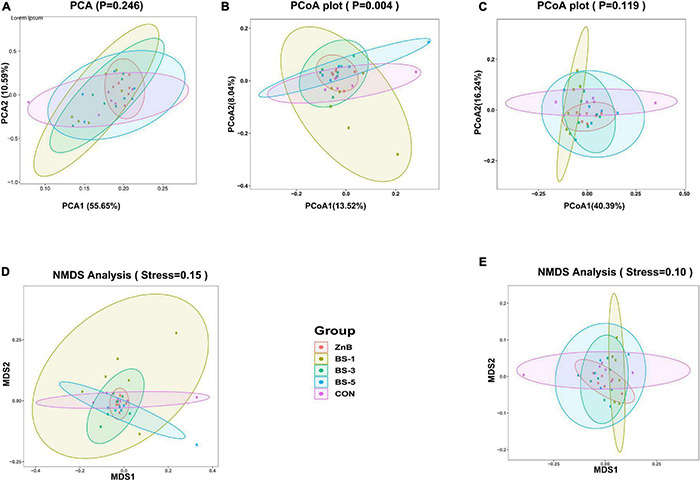
Effect of dietary supplementation of *Bacillus subtilis* ATCC19659 on cecal microbiota communities of broilers by beta diversity measures. PCA **(A)**, weighted PCoA **(B)**, unweighted PCoA **(C)**, weighted NMDS **(D)**, and unweighted NMDS **(E)**. CON, chicks were fed a corn-and soybean-based diet; ZnB, chicks were fed basal diet containing 500 mg ZnB/kg; BS-1, chicks were fed basal diet containing 1 × 10^8^ CFU/g feed of *BS*-ATCC19659; BS-3, chicks were fed basal diet containing 3 × 10^8^ CFU/g feed of *BS*-ATCC19659; BS-5, chicks were fed basal diet containing 5 × 10^8^ CFU/g feed of *BS*-ATCC19659.

The alpha diversity indicators of observed OUT, Chao1, Shannon, Simpson, and Goods coverage indices among the treatment groups are presented in Table 6. The results of indices as affected by the dietary inclusion of ZnB and *BS*-ATCC19659 showed no significant effect (*p* > 0.05) on alpha diversity indicators (Table 6). Beta diversity indicators of PCA, PCoA, and NMDS measured the intragroup and intergroup distances and are presented in Figure 2. Statistically significant *p*-values measured the intragroup and intergroup distances and found that the differences between groups were not significant plots of PCA (Figure 2A), unweighted PCoA (Figure 2C), and weighted and unweighted NMDS (Figures 2D,E), while a significant difference was estimated in the weighted PCoA plot (*p* < 0.05) (Figure 2B).

**TABLE 6:** Effect of dietary supplementation of *Bacillus subtilis* ATCC19659 on cecal microbiota communities of broilers by alpha diversity measures.

Items	Treatments^a^					SEM	*p*-value
	CON	ZnB	BS-1	BS-3	BS-5		
Observed OTU	486.50	521.67	507.67	519.33	476.50	16.689	0.901
Chao1	525.62	558.98	555.19	557.43	506.12	18.687	0.881
Shannon	6.62	6.98	6.75	6.97	6.83	0.085	0.661
Simpson	0.97	0.98	0.97	0.98	0.98	0.003	0.618
Goods coverage	1	1	1	1	1	0.001	0.947

*^a^CON, chicks were fed a corn-and soybean-based diet; ZnB, chicks were fed basal diet containing 500 mg ZnB/kg; BS-1, chicks were fed basal diet containing 1 × 10^8^ CFU/g feed of BS-ATCC19659; BS-3, chicks were fed basal diet containing 3 × 10^8^ CFU/g feed of BS-ATCC19659; BS-5, chicks were fed basal diet containing 5 × 10^8^ CFU/g feed of BS-ATCC19659.*

### Microbial Composition Assessment of Host Broiler

**FIGURE 3:**
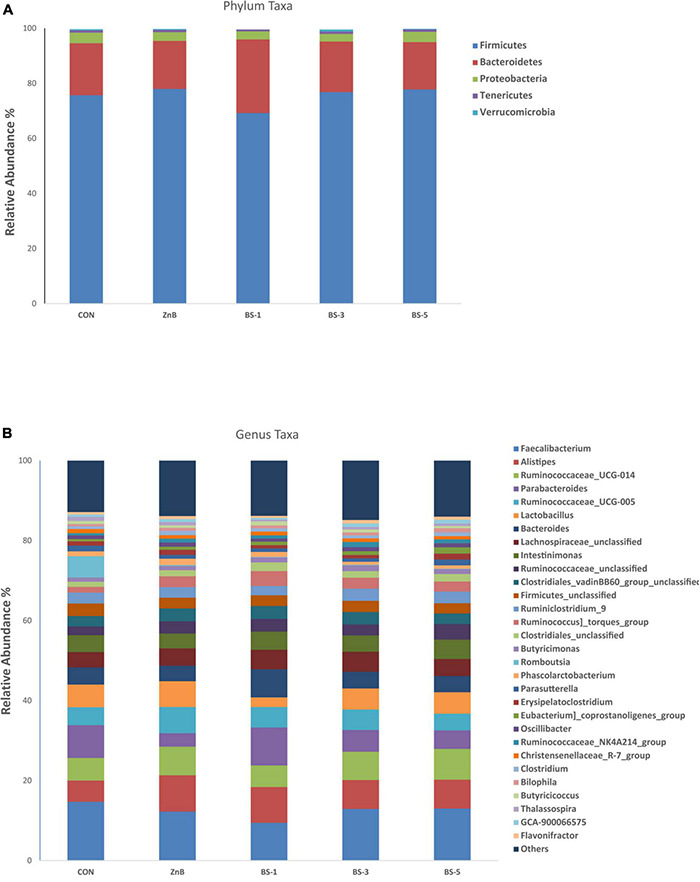
Effect of dietary supplementation of *Bacillus subtilis* ATCC19659 on the relative abundance of cecal microbiota communities at phylum **(A)** and genus **(B)**. CON, chicks were fed a corn- and soybean-based diet; ZnB, chicks were fed basal diet containing 500 mg ZnB/kg; BS-1, chicks were fed basal diet containing 1 × 10^8^ CFU/g feed of *BS*-ATCC19659; BS-3, chicks were fed basal diet containing 3 × 10^8^ CFU/g feed of *BS*-ATCC19659; BS-5, chicks were fed basal diet containing 5 × 10^8^ CFU/g feed of *BS*-ATCC19659.

The effect of the different levels of *BS*-ATCC19659 groups as a dietary supplement on different phylum and genus taxa levels in the cecal microbial composition is shown in Figure 3. The analysis of the phylum level revealed that *Firmicutes* and *Bacteroidetes* were the predominant phyla of the cecal community, accounting for approximately 75.5 and 19.7%, respectively (Figure 3A). The relative abundance of *Cyanobacteria* phyla was significantly decreased in the ZnB and BS-1 groups compared to the CON group (*p* < 0.05), as shown in Table 7.

**TABLE 7:** Effect of dietary supplementation of Bacillus subtilis ATCC19659 on bacterial taxonomy within the cecal contents of broilers.

Items	Relative abundance (%)					SEM	*p*-value
	^1^CON	ZnB	BS-1	BS-3	BS-5		
**Phylum**							
*Cyanobacteria*	0.1^a^	0.02^b^	0.02^b^	0.08^ab^	0.03^b^	0.01	0.047

**Genus**							
*CHKCI001*	0.51^a^	0.19^b^	0.13^b^	0.29^ab^	0.18^b^	0.038	0.02
*GCA-900066575*	0.54^b^	0.75^ab^	0.48^b^	0.87^a^	0.93^a^	0.05	0.01
*Anaerofustis*	0.005^a^	0.004^ab^	0^b^	0.006^a^	0.009^a^	0.001	0.03
*Papillibacter*	0.019^ab^	0.032^a^	0.007^b^	0.039^a^	0.031^a^	0.004	0.03
*Escherichia–Shigella*	0.64^a^	0.36^ab^	0.82^a^	0.09^b^	0.23^ab^	0.11	0.04
*Clostridia_unclassified*	0.01^a^	0^b^	0.006^ab^	0.002^b^	0^b^	0.002	0.04
*Lactobacillus*	5.66^a^	6.45^a^	2.40^b^	5.25^a^	5.37^a^	0.71	0.04

*^1^CON, chicks were fed a corn-and soybean-based diet; ZnB, chicks were fed basal diet containing 500 mg ZnB/kg; BS-1, chicks were fed basal diet containing 1 × 10^8^ CFU/g feed of BS-ATCC19659; BS-3, chicks were fed basal diet containing 3 × 10^8^ CFU/g feed of BS-ATCC19659; BS-5, chicks were fed basal diet containing 5 × 10^8^ CFU/g feed of BS-ATCC19659.*

*^a,b,c^Values in the same row with different letter superscripts mean significant differences (p < 0.05). Data are presented as the mean ± SEM (n = 6).*

Regarding genus taxa level, *Faecalibacterium*, *Alistipes*, and *Ruminococcaceae*_UCG-014 were the dominant genera of the cecal community, accounting for approximately 12.46, 7.54, and 6.59%, respectively (Figure 3B). The relative abundance of the *CHKCI001* genus was significantly increased in the CON group as compared to the ZnB and *BS*-ATCC19659 groups (*p <* 0.05). The relative abundance of *GCA-900066575* (*Lachnospiraceae*) was significantly increased in the BS-5 and BS-3 groups compared to the CON group (*p* < 0.05); the relative abundance of *Papillibacter* was significantly higher in the BS-3 and ZnB groups than in the CON group (*p* < 0.05). However, the relative abundance of *Anaerofustis* was significantly lower in the BS-1 and ZnB groups than in other groups (*p* < 0.05). The relative abundance of *Escherichia–Shigella* was significantly reduced in the BS-3 group as compared to other treatments (*p* < 0.05); the relative abundance of *Clostridia_unclassified* was significantly higher in CON (*p* < 0.05) than in other treatments. The relative abundance of *Lactobacillus* in the BS-1 group significantly decreased compared with the other treatments (*p* < 0.05). However, the ZnB group tended to increase in the relative abundance of *Lactobacillus*, as shown in Table 7.

## Discussion

In the present study, it was found that adding 3 and 5 × 10^8^ CFU of *BS*-ATCC19659/g feed for 42 days helped improve broiler performance (growth performance, antioxidant status, immunity, and gastrointestinal responses) by colonizing a distinct GM community in the gastrointestinal tract, resulting in improved gut health for broiler productivity. These findings could be a suitable alternative to the use of ZnB antibiotics in organic poultry production. Precisely, probiotic products are widely used in several commercial applications, especially *Bacillus subtilis* strains that support digestion, healthy GM, and immune response, as an alternative to an antibiotic in poultry diets (Gao et al., 2017; Qin et al., 2018).

In the present study, BS-5 and BS-3 groups improved body weight at the 21^st^ and 42^nd^ days and ADG during 1–21 days. They showed consistency with the findings of Bai et al. (2017), who tested the inclusion of *Bacillus subtilis* and found an increase in broiler growth performance, providing beneficial effects on broiler growth and potentially replacing antibiotic growth promoters (Park et al., 2020). In broiler chickens, some certain strains of *Bacillus subtilis* such as *Bacillus subtilis* B10 (Qin et al., 2018), *Bacillus subtilis* 29784 (Jacquier et al., 2019), *Bacillus subtilis* DSM 32315 (Bortoluzzi et al., 2018), *Bacillus subtilis* BYS2 (Dong et al., 2020), *Bacillus subtilis* GM5 (Hadieva et al., 2021), and *Bacillus subtilis* PB6 (Abramowicz et al., 2019) have been suggested to promote growth activities and healthy GM. The expression of mRNA *GHR* and mRNA *IGF-1* were increased in the *BS*-ATCC19659 (BS-1 and BS-3) groups in the liver. *IGF-1* and *GHR* genes play a critical role in the growth rate of chicken. Thus, the improved effect of the BS-3 group may be linked to the alteration in genes in broiler-related growth (Kareem et al., 2016). However, other researchers illustrated that dietary supplementation of probiotics sometimes had minimal or no effect on growth performance. This may be due to strains of probiotic bacteria, preparation methods, administration, dosage, composition, hygiene status, and bird age (Lee et al., 2010; Zhang et al., 2012).

The shift in balance between oxidants [such as reactive oxygen species (ROS) and free radicals] and antioxidants in favor of oxidants is termed “oxidative stress.” Oxidative stress severely affects Broiler chickens, damaging nucleic acids, cell molecules, and mitochondrial membranes. Several studies reported that additional probiotics containing *Bacillus* spp. in diet enhanced oxidative resistance and increased exogenous antioxidant capacity in broiler chickens (Bai et al., 2017; Abramowicz et al., 2019; Dong et al., 2020). Both BS-5 and ZnB groups had a significant increase in GSH, T-AOC, CAT, and SOD activities from the results obtained. They decreased the MDA concentration in the entire period in serum at 42 days. The relative mRNA expressions of antioxidant genes including *SOD*, *CAT*, and *GPX* were significantly increased in *BS*-ATCC19659 groups (BS-1 and BS-5) in liver tissue. The results of Zhang et al. (2017) were in agreement that the dietary *Bacillus species* in broilers improved significantly in both GSH, SOD, and CAT activities in serum. Likewise, dietary *Bacillus subtilis* improved the antioxidant capacity in broiler chickens (Dong et al., 2020). He et al. (2019) demonstrated that probiotics had a positive role in oxidation resistance, scavenging ROS and stimulating antioxidant capability. The animal defense system can depend on probiotics as a natural source for protection of the oxidative stress result from ROS.

Immunoglobulins and complement components are parameters that reflect the immune status of animals because it plays crucial roles in the immune system (Rajput and Li, 2012). The application of probiotics stimulates immune cells and cytokine production. Our findings revealed that BS-5 and ZnB groups improved serum immunoglobulins (IgM, IgA, and IgE), secretory IgA (sIgA), and complement components (C3 and C4). The results agree with Bai et al. (2017), who reported that *Bacillus subtilis* fmbJ increased the serum IgA and IgG concentrations of broiler chicken at 42 days. Also, Sun et al. (2011) reported that serum C4 and C3 were increased with the addition of *Psychrobacter* spp. in the broiler diet. Likewise, the *Bacillus subtilis* probiotic increased the serum sIgA and lgA of broiler chickens (Rajput et al., 2017). sIgA works on the sustenance of mucosal homeostasis that influences the intestinal microbiota content and improves the immune system (Lammers et al., 2010; Wang et al., 2017; Liu et al., 2019). In the present study, serum interleukin (IL-10) and tumor necrosis factor-beta (TGF-β) were enhanced in BS-5 and ZnB groups, but serum IL-6, IL-4, and TNF-α were increased in the CON group and BS-1 groups. The mRNA expressions of *TGF-β* and *IFN-γ* were significantly increased with the BS-3 group in liver tissue. IFN-γ is a standard indicator of cellular immunity through the higher levels and has been linked with enhanced protective immune (Lee et al., 2014). TGF-α and IL-10 stimulate class switching of B cells to create IgA that proliferates and activates by interactions with T cells (Bos et al., 2001; Azad et al., 2018). This result agrees with Selvam et al. (2009) who observed that the inclusion of *Bacillus subtilis* PB6 had significantly higher levels of anti-inflammatory cytokines (TGF-β and IL-10) in plasma and significantly decreased the levels of pro-inflammatory cytokines (IL-6 and TGF-α) because it is found to secrete surfactins of cyclic lipopeptides (antibacterial). These surfactins prevent phospholipase A2. The phospholipase A2 is a rate-limiting enzyme that takes part in the arachidonic acid linked with the inflammatory pathway. Conversely, Zhang et al. (2016) discovered that supplementation of *Clostridium butyricum* changed the sensitization of the host by increasing the concentrations of IL-6, IL-8, and TNF-α. Also, Rajput et al. (2013) reported that dietary supplementation with *Bacillus subtilis* increased IL-6 and TFN-α compared to a control group in the ileum and jejunum. Huang et al. (2012) reported an increase in the concentration of inflammatory cytokines. These might be due to different probiotics species.

The dietary *Bacillus subtilis* supplementation enhances the absorption surface because it improves the crypt cell proliferation in the small intestinal and *Bacillus* spp. can also colonize and form niches in the intestine protected from pathogens and increase villus growth (Li et al., 2019). This result agrees with Deng et al. (2012), who reported that *Bacillus licheniformis* of dietary supplementation significantly increased the villus length/crypt depth ratio of the ileum at 6 days. In addition, *Bacillus coagulans* TBC169 significantly increased the villus length/crypt depth ratio of the jejunum (21^st^ and 42^nd^ days) and ileum (42 days) (Li et al., 2019). Thus, *Bacillus* spp. might improve the absorptive surface of the jejunum and ileum, which is attributed to the developing proliferation of crypt cells in the small intestine and improves the growth performance in the broiler (He et al., 2019).

The indexes of Simpson and Shannon express a diversity of the microbiome community, while the chao1 index and observed species can reflect the richness of microbial diversity (Hagerty et al., 2020). PCA, PCoA, and NMDS analysis expressed that the antibiotic (ZnB) and *BS*-ATCC19659 groups were similar in the current experiment, and this result is in agreement with Hong et al. (2019) who reported that the NMDS was similar between the antibiotic group (chlortetracycline) and *Bacillus amyloliquefaciens* TL group. However, Ma et al. (2018) found that PCoA analysis separated community microbes between groups (control and *Bacillus subtilis* DSM 32315), and PCA analysis explained 33% of microbial diversity.

The intestinal microbiota plays several roles in intestinal morphology, immunity, nutrient absorption and metabolism, and host health (Hong et al., 2019). In this study, *Firmicutes* and *Bacteroidetes* were the predominant phylum, accounting for more than 90% of chickens. It is worth stating that some studies indicated that the dominant phylum of the cecal community is *Firmicutes* and *Bacteroidetes* in chickens (Danzeisen et al., 2011; Wei et al., 2013; Shaufi et al., 2015). Nevertheless, the dominant bacteria may change at the phylum level because of breed, age, and regional variances of selected chickens. The relative abundance of *Cyanobacteria* phyla decreased in ZnB and BS-1 groups compared to the CON group. The result of Trela et al. (2020) agrees that the inclusion of *Bacillus licheniformis* decreased *Cyanobacteria* in cecal content microbiota. *Cyanobacteria* phyla had some species that can produce some neurotoxins that cause diseases (Codd et al., 2005). *Bacillus* spp. enhanced the immunological intestinal mucosa function and regulated the bacterial flora in the intestine (Li et al., 2019).

According to our result, *Faecalibacterium*, *Alistipes*, and *Ruminococcaceae_UCG-014* were the major bacterial genera in the cecum community. This result agrees with Xiao et al. (2017) and Zhu et al. (2020), who reported that *Alistipes*, *Ruminococcin*, and *Faecalibacterium* were the predominant genus of bacteria in the cecal community. The relative abundance of genera *GCA-900066575* (*Lachnospiraceae*), *Anaerofustis*, and *Papillibacter* tended to increase in *BS*-ATCC19659 groups. These genera belong to *Firmicutes*, which is associated with increased weight gain in chickens by producing molecules that absorb them from the host gut wall directly as a source of energy (Chen and Yu, 2020; Adewole and Akinyemi, 2021; Dauksiene et al., 2021). Similarly, Jumpertz et al. (2011) stated that increasing the rate of nutritional absorption in feces was associated with increased Firmicutes. In this study, the proliferation of potentially pathogenic bacteria and the relative abundance of the genera *Escherichia_Shigella* and *Clostridia_unclassified* were inhibited by ZnB and *BS*-ATCC19659 (BS-3, and BS-5) groups in the cecal community. Consistent with our results, Li et al. (2020) reported that fermented soybean meal used in broiler diet significantly reduced the relative abundance of genera *Escherichia–Shigella* and *Clostridiales*. *Escherichia–Shigella* as opportunistic pathogenic bacteria were described to destroy the intestinal structure and estimate pro-inflammatory activities by variable ways such as propagating the virulence factors, leading to an increase in the risk of infection and diarrhea of host (Ma et al., 2018). *Clostridia_unclassified* can compete and interact with other microbiota for proliferation in the intestine. *Clostridia_unclassified* produces some toxins that cause serious poultry diseases (Bortoluzzi et al., 2019). *Bacillus* spp. create bacteriocins, which have been stated to display antibacterial function in various models (Riazi et al., 2009; Nobutani et al., 2017). Meanwhile, *Bacillus* spp. produce is related to lactic acid as well as other organic acid production. Lactic acid can suppress gut colonization of harmful bacteria (Cui et al., 2005). Recently, *Bacillus* spp. produced dysprosium, which has been identified to display a broad antibacterial spectrum (Li et al., 2019). Also, *Bacillus* spp. can maintain the balance of GM *via* converting polysaccharides to oligosaccharides (Zheng et al., 2011). The relative abundance of genus *lactobacillus* improved among treatments when compared to the BS-1 group. *Lactobacillus* is one of the essential probiotic bacteria because it improves growth performance and helps maintain the microbial balance in the intestine, suppresses harmful pathogens, and stimulates the growth of beneficial bacteria (Shokryazdan et al., 2017).

## Conclusion

In conclusion, dietary supplementation with the BS-5 group (*Bacillus subtilis* ATCC19659 5 × 10^8^ CFU/g feed) significantly increased bodyweight, improved antioxidant capacity, enhanced immune response, and absorption of nutrition in the present experiment. Furthermore, *Bacillus subtilis* ATCC19659 affected cecal microbial composition. Therefore, we conclude that feeding a probiotic additive *Bacillus subtilis* ATCC19659 in the poultry industry might be an encouraging alternative to antibiotic growth promoters.

## Data Availability Statement

The 16sRNA sequencing raw data is available at NCBI by accession number PRJNA807482 https://www.ncbi.nlm.nih.gov/bioproject/?term=PRJNA807482. The rest of the raw data supporting the conclusions of this article will be made available by the authors, without undue reservation.

## Ethics Statement

The License of Experimental Animals (SYXK 2014-0002) of the Animal Experimentation Ethics Committee of Southwest University, Chongqing, China, and birds were raised following the guidelines described by the Animal Care Committee of Chongqing, China. In addition, efforts were made to reduce animal suffering and were carried out in compliance with the “ARRIVE” guidelines for reporting *in vivo* experiments in animal research.

## Author Contributions

TM: investigation, data curation, methodology, formal analysis, and writing—original draft. WS and GB: investigation and methodology. AE, KM, and RZ: writing—review and editing. PH and LW: conceptualization and supervision. ZT: investigation, project administration, funding acquisition, and writing—reviewing and editing. All authors contributed to the article and approved the submitted version.

## Conflict of Interest

The authors declare that the research was conducted in the absence of any commercial or financial relationships that could be construed as a potential conflict of interest.

## Publisher’s Note

All claims expressed in this article are solely those of the authors and do not necessarily represent those of their affiliated organizations, or those of the publisher, the editors and the reviewers. Any product that may be evaluated in this article, or claim that may be made by its manufacturer, is not guaranteed or endorsed by the publisher.

## Footnotes

1
http://www.njjcbio.com/
