# Supplementary material for: Feeding Bacillus subtilis ATCC19659 to Broiler Chickens Enhances Growth Performance and Immune Function by Modulating Intestinal Morphology and Cecum Microbiota
Source: Front Microbiol. 2022 Feb 22;12:798350. doi: 10.3389/fmicb.2021.798350 (PMC8904210; doi:10.3389/fmicb.2021.798350)
Supplement: Supplementary file 1 [file Data_Sheet_1.docx]

**Supplementary data**

**Table S1**. The information of all kits used to measure blood biochemical in serum chicken

| **Kit name/unit** | **Description** | **Cat. No.** |
| --- | --- | --- |
| SOD (u/ml) | Total Superoxide Dismutase (T-SOD) kit  (Hydroxy. method) | A001-1-2 |
| CAT (U/ml) | Catalase (CAT) assay kit (Visible light) | A007-1-1 |
| MDA(nmol/ml) | Malondialdehyde assay kit | A003-1-3 |
| T-AOC (U/ml) | Total antioxidant capacity assay kit | A015-1-2 |
| GSH (mg/gl) | Reduced glutathione (GSH) assay kit (Spect. method) | A006-1-1 |
| IL-10 (ng/L) | Interleukin -10 Assay Kit | H009 |
| TNF-α (ng/L) | Tumor Necrosis Factor-α Assay Kit | H052 |
| IL-4 (ng/L) | Interleukin -4 Assay Kit | H005 |
| IL-6 (ng/L) | Interleukin -6 Assay Kit | H007 |
| TGF-β (ng/L) | Transforming growth factor-β Assay Kit | H034 |
| IgE(ug/ml) | Immunoglobulin E Assay Kit | H107 |
| IgA(ug/ml) | Immunoglobulin A Assay Kit | H108 |
| IgG (ug/ml) | Immunoglobulin G Assay Kit | H106 |
| IgM (ug/ml) | Immunoglobulin M Assay Kit | H109 |
| C3 (mg/mL) | Complement 3 Assay Kit | H186-1 |
| C4 (mg/mL) | Complement 4 Assay Kit | H186-2 |
| sIgA (ng/ml) | Secretory Immunoglobulin A ELISA kit | H108-2 |
| Pepsin (U/ml) | Pepsin assay kit | A080-1-1 |
| Lipase (U/l) | Lipase assay kit | A054-1-1 |
| Amylase(U/dl) | α-Amylase Assay Kit | C016-1-1 |

All commercial kits purchased from Nanjing Jiancheng Bioengineering Institute, Nanjing, China (NJJCBIO, *http://www.njjcbio.com/*)

**Table S2.** Sequences of the oligonucleotide primers used for quantitative real-time PCR

| Accession number | Primer sequence (5-3) | Gene name |
| --- | --- | --- |
| NM_205427.1 | F:- AACGCCAAAGCCTCAAC  R:- TGAGGTGAAGGTTGCGAGGC | ***IFN-****γ* |
| NM_205454.1 | F:-CGGGACGGATGAGAAGAAC  R:- CGGCCCACGTAGTAAATGAT | ***TGF-β*** |
| NM_205064.1 | F:- GCACGGTGGACCAAAAGA  R:- AACGAGGTCCAGCATTTCC | *SOD* |
| NM_001031215.2 | F:-GGTTCGGTGGGGTTGTCTTT  R:- CACCAGTGGTCAAGGCATCT | *CAT* |
| NM_001277853.1 | F:-CAGGAGAACGCCACCAACG  R:- TCTCAGGAAGGCGAACAGC | *GPX* |
| NM_001001293.1 | F:- AACACAGATACCCAACAGCC  R:- AGAAGTCAGTGTTTGTCAGGG | *GHR* |
| NM_001004384.2 | F:- CATTCATTTCTTCTACCTTGGC  R:-AGCAGCATTCATCCACTATTCC | *IGF-1* |
| NM_205518.1 | F:- GAGAAATTGTGCGTGACATCA  R:- CCTGAACCTCTCATTGCCA | *β-actin* |

Abbreviation: interferon γ (IFN-γ); transforming Growth Factor (TGF-β); Superoxide dismutase (SOD); Catalase (CAT); glutathione peroxidase (GPX); growth hormone receptor (GHR) and insulin like growth factor 1 (IGF-1).
